# Pain and mental health - separate and joint associations with sickness absence among young employees

**DOI:** 10.1093/eurpub/ckac129.310

**Published:** 2022-10-25

**Authors:** P Fagerlund, R Shiri, J Suur-Uski, S Kaartinen, O Rahkonen, T Lallukka

**Affiliations:** 1 Department of Public Health, University of Helsinki, Helsinki, Finland; 2 Finnish Institute of Occupational Health, Helsinki, Finland; 3 Department of Physical and Rehabilitation Medicine, Helsinki University Hospital, Hyvinkää, Finland

## Abstract

**Background:**

Both pain and mental illness associate with work disability. However, few studies have examined the association of concurrent pain and mental distress with sickness absence (SA). We examined separate and joint associations of chronic pain, multisite pain, and mental distress with total and long-term all-cause SA among young and midlife municipal employees.

**Methods:**

As part of the Young Helsinki Health study, baseline data were collected in 2017 from 19-39-year-old employees of the City of Helsinki, Finland. Chronic (≥3 months) pain, multisite (≥2 body sites) pain and mental distress (RAND-36 emotional wellbeing subscale below median) were reported by 3911 respondents. Register data on total (>1 day) and long-term ((>11 workdays) SA for the following year were obtained from the employer and the Social Insurance Institute of Finland with respondents’ informed consent. Negative binomial regression analyses were performed with sociodemographic, socioeconomic, and health-related factors as confounders. The interaction of gender was examined.

**Results:**

Chronic pain, multisite pain, and mental distress were associated with total SA. Chronic multisite pain was associated with long-term SA (rate ratio [RR] 2.51, 95% CI 1.17-5.42), and chronic pain (RR 5.04, 95% CI 2.14-11.87) and multisite pain (RR 4.88, 95% CI 2.30-10.33) with long-term SA among those with mental distress. For women, there was a synergistic interaction of multisite pain to the association with total SA (synergy index 1.80, 95% CI 1.27-2.54).

**Conclusions:**

Chronic and multisite pain associate with SA among young and midlife employees. The associations are generally stronger among women and particularly among those with concurrent mental distress. Interventional studies are needed to confirm if early symptom recognition and support could reduce sickness absence.

**Key messages:**

• Chronic pain and pain at multiple body sites associate with sickness absence among young and midlife employees, particularly among women and those with concurrent mental distress.

• Interventional studies are needed to confirm if sickness absence could be reduced by early recognizing pain and mental distress among employees and providing preventive and therapeutic services.

